# Antimicrobial Lemongrass Essential Oil—Copper Ferrite Cellulose Acetate Nanocapsules

**DOI:** 10.3390/molecules21040520

**Published:** 2016-04-20

**Authors:** Ioannis L. Liakos, Mohamed H. Abdellatif, Claudia Innocenti, Alice Scarpellini, Riccardo Carzino, Virgilio Brunetti, Sergio Marras, Rosaria Brescia, Filippo Drago, Pier Paolo Pompa

**Affiliations:** 1 Smart Materials Group, Nanophysics Department, Istituto Italiano di Tecnologia (IIT), Via Morego 30, 16163 Genoa, Italy; Riccardo.Carzino@iit.it; 2 Nanostructures Department, Istituto Italiano di Tecnologia (IIT), Via Morego 30, 16163 Genoa, Italy; Mohamed.Abdellatif@iit.it; 3 INSTM-RU of Florence and Department of Chemistry, University of Florence, Via Della Lastruccia 3-13, 50019 Sesto F.no, Firenze, Italy; claudia.innocenti@unifi.it; 4 Nanochemistry Department, Istituto Italiano di Tecnologia (IIT), Via Morego 30, 16163 Genoa, Italy; Alice.Scarpellini@iit.it (A.S.); Sergio.Marras@iit.it (S.M.); Rosaria.Brescia@iit.it (R.B.); Filippo.Drago@iit.it (F.D.); 5 Biomolecular Nanotechnologies, Istituto Italiano di Tecnologia (IIT), Via Barsanti 1, 73010 Lecce, Italy; Virgilio.Brunetti@iit.it; 6 Nanobiointeractions & Nanodiagnostics, Istituto Italiano di Tecnologia (IIT), Via Morego 30, 16163 Genoa, Italy; Pierpaolo.Pompa@iit.it

**Keywords:** antimicrobial nanocapsules, lemongrass essential oil, copper ferrite nanoparticles, cellulose acetate, drug delivery, antimicrobial resistance

## Abstract

Cellulose acetate (CA) nanoparticles were combined with two antimicrobial agents, namely lemongrass (LG) essential oil and Cu-ferrite nanoparticles. The preparation method of CA nanocapsules (NCs), with the two antimicrobial agents, was based on the nanoprecipitation method using the solvent/anti-solvent technique. Several physical and chemical analyses were performed to characterize the resulting NCs and to study their formation mechanism. The size of the combined antimicrobial NCs was found to be *ca.* 220 nm. The presence of Cu-ferrites enhanced the attachment of LG essential oil into the CA matrix. The magnetic properties of the combined construct were weak, due to the shielding of Cu-ferrites from the polymeric matrix, making them available for drug delivery applications where spontaneous magnetization effects should be avoided. The antimicrobial properties of the NCs were significantly enhanced with respect to CA/LG only. This work opens novel routes for the development of organic/inorganic nanoparticles with exceptional antimicrobial activities.

## 1. Introduction

Cellulose acetate (CA) is a biopolymer prepared through the reaction of cellulose with acetic anhydride and acetic acid in the presence of sulfuric acid [1]. Cellulose acetate has many applications in the pharmaceutical field, including drug or enzyme delivery [1],wound dressings of CA nanofibers with essential oils [2], chlorinated CA with modified 4,4′-diphenylmethane diisocyanate nanofiber mats with antimicrobial properties [3], electrospun CA with other polysaccharide nanofibers [4] with biomedical properties, antifouling coatings of CA thin films with polysaccharide multilayers [5] and others. During the last few years CA nanoparticles have been formed [6] and incorporated with chitosan, which has antimicrobial properties [7]. Recently, pure CA nanocapsules (NCs) loaded with natural antimicrobial agents, such as essential oils, have been prepared with considerable antimicrobial efficiency [8]. Essential oils are well known for their antimicrobial properties [9] and for being kept active, even if encapsulated into polymers [2,10,11].

Magnetic nanoparticles, such as iron oxide nanoparticles, have been found to be effective in fighting infectious diseases [12,13,14,15]. Transition metals of copper, zinc, chromium and nickel have been substituted into cobalt ferrite nanoparticles and have shown very good antimicrobial properties [16]. Moreover, silver nanoparticles loaded into copper ferrite (CuFe_2_O_4_) magnetic hollow fibers showed excellent antimicrobial efficacy against four bacteria: *E. coli*, *S. typhi*, *S. aureus* and *V. parahaemolyticus* [17].

Ferrites are semiconductor ceramics with the general formula of spinel structure AB_2_O_4_, where A and B represent various metal cations, including iron. Ferrites are considered a class of spinels that consist of cubic closed pack oxides with A cations occupying 1/8th of the octahedral sites, and B cations occupying half of the octahedral sites. For the inverse spinel structure, half of the B cations occupy tetrahedral sites, and both the A and B cations occupy the octahedral sites. Meanwhile, divalent and trivalent cations can occupy both A and B sites [18,19,20]. Cu-ferrite is known to have non-collinear saturation magnetization, due to dilution of the magnetic properties with Cu ions [21].

Some kind of ferrites, such as Cu-ferrites, are of great interest as soft magnets, since they can be magnetized, de-magnetized and their magnetic properties controlled through encapsulation. Some researchers suggested that substitution of spinel iron oxide with metals can help in enhancing the biomedical properties of the ferrite nanoparticles [16,22]. 

In this work, we studied the effect of substituting the spinel iron oxide by Cu ions to form Cu-ferrite with low copper content. Exploiting the nontoxicity of Cu-ferrites [23], we combined copper ferrite of the spinel structure, CuFe_2_O_4_, prepared by the co-precipitation method, with CA/lemongrass (LG) NCs to improve their antimicrobial activity. The Cu-ferrite nanoparticles considerably enhanced the antimicrobial activity of CA/LG essential oil NCs and showed complete block of bacteria growth. The final nanocomposite structure of CA/LG/Cu-ferrite NCs showed significant enhancement in the antimicrobial properties compared to the previous study of CA/LG NCs alone [8].

## 2. Results and Discussion

### 2.1. Dynamic Light Scattering

**Figure 1:**
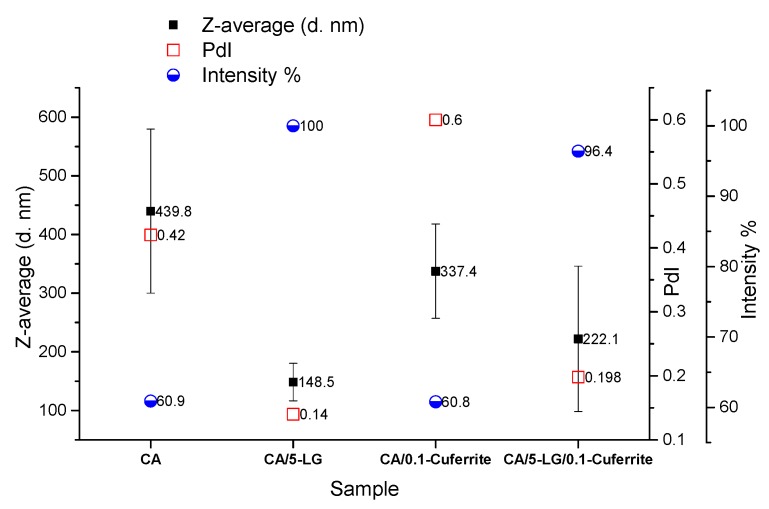
DLS measurements of the size, polydispersity index (PdI) and intensity % of the Cellulose acetate (CA) nanoparticles, CA/5-lemongrass (LG), CA/Cu-ferrite and CA/5-LG/Cu-ferrite nanocapsules (NCs).

DLS was used to determine the diameter of the formed nanoparticles and nanocapsules. It was found that the addition of either Cu-ferrite NPs or LG essential oil lowered the size of the CA NPs (440 nm) (Figure 1). The size of the CA/Cu-ferrite NCs was 340 nm, whereas the size of the CA/5-LG NCs was 150 nm (Figure 1). By adding both antimicrobial agents, the overall size of the CA/5-LG/Cu-ferrite NCs was about 225 nm (Figure 1), higher than the CA/5-LG NCs (150 nm) and bare Cu-ferrite NPs (70 nm), as will be shown later in the SEM section, revealing that Cu-ferrite increased the size of the CA/5-LG NCs. The polydispersity index (PdI) of the CA/5-LG and CA/5-LG/Cu-ferrite NCs with the presence of LG was low, whereas the PdI index of CA NPs and CA/Cu-ferrite NCs was high, revealing that LG essential oil helped to stabilize the formation of the resulting NCs, as shown in Figure 1. Similarly, and for the same reason, the intensity % is higher when LG essential oil was present. The reason that the Cu-ferrite and LG oil lowers the diameter of bare CA was due to the reaction between them and the consequent encapsulation, as will be demonstrated later, making them more stable than bare CA NPs.

### 2.2. X-ray Diffraction

**Figure 2:**
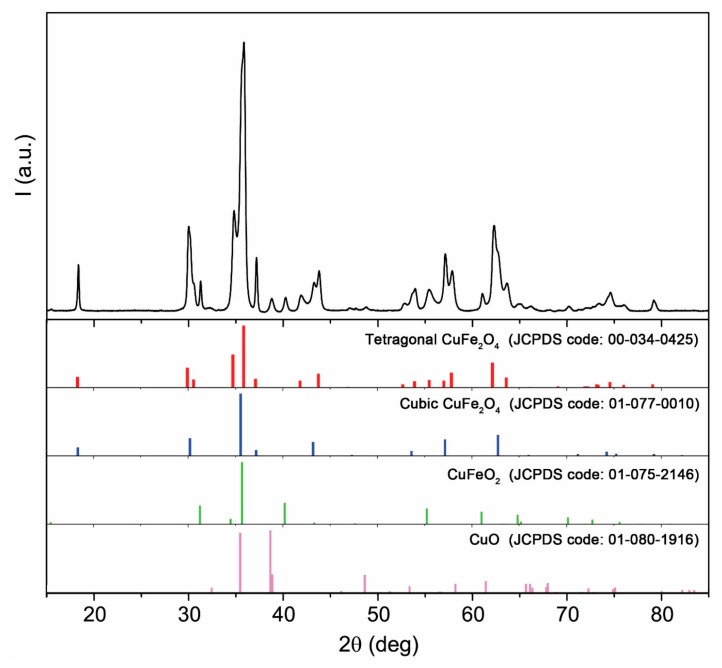
XRD pattern of Cu-ferrite NPs.

The bare Cu-ferrite NPs, investigated via X-ray diffraction (XRD), consist of a mixture of four different crystalline phases, as shown in Figure 2. Semi-quantitative analysis based on the reference intensity ratio (RIR) was carried out to estimate the amount of all of the identified phases, with the following results: 66 wt % tetragonal CuFe_2_O_4_ (copper ferrite, space group: I41/amdS(141)), 24 wt % cubic CuFe_2_O_4_ (cuprospinel, space group: Fd-3m(227)), 6 wt % CuFeO_2_ (delafossite, space group: R-3m(166)) and 4 wt % CuO (tenorite, space group: Cc(9)). XRD analysis on CA/5-LG/Cu-ferrite NCs was mainly covered from the CA polymers, and it was not easy to see the Cu-ferrite NPs due to their small concentration below the sensitivity of the technique (*i.e.*, below 1%).

### 2.3. Atomic Force Microscopy

**Figure 3:**
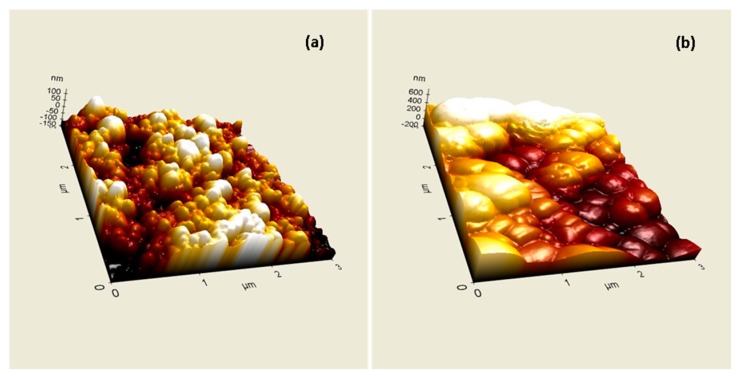
AFM topography of (**a**) CA/5-LG NCs and (**b**) CA/5-LG/Cu-ferrite NCs.

Atomic force microscopy was used to reveal the topographical differences with the addition of Cu-ferrite NPs in the nanocapsules. As was observed from Figure 3a, CA/5-LG NCs are spherical in shape and have diameters less than 200 nm, as was also proven previously by DLS measurements. When Cu-ferrite was added to the NC, such as the CA/5-LG/Cu-ferrite NCs, the NCs were no longer spherical and resemble more the shapes of Cu-ferrites, *i.e.*, tetragonal and cubic, as shown in Figure 3b. Furthermore, the 3D topography in Figure 3b reveals that the CA/5-LG/Cu-ferrite NCs were larger in size than the NCs without Cu-ferrite (Figure 3a), reaching sizes of more than 200 nm, in accordance with the DLS measurements shown in Figure 1.

### 2.4. High Resolution Scanning Electron Microscopy, Energy Dispersive X-ray Spectroscopy and Transmission Electron Microscopy

**Figure 4:**
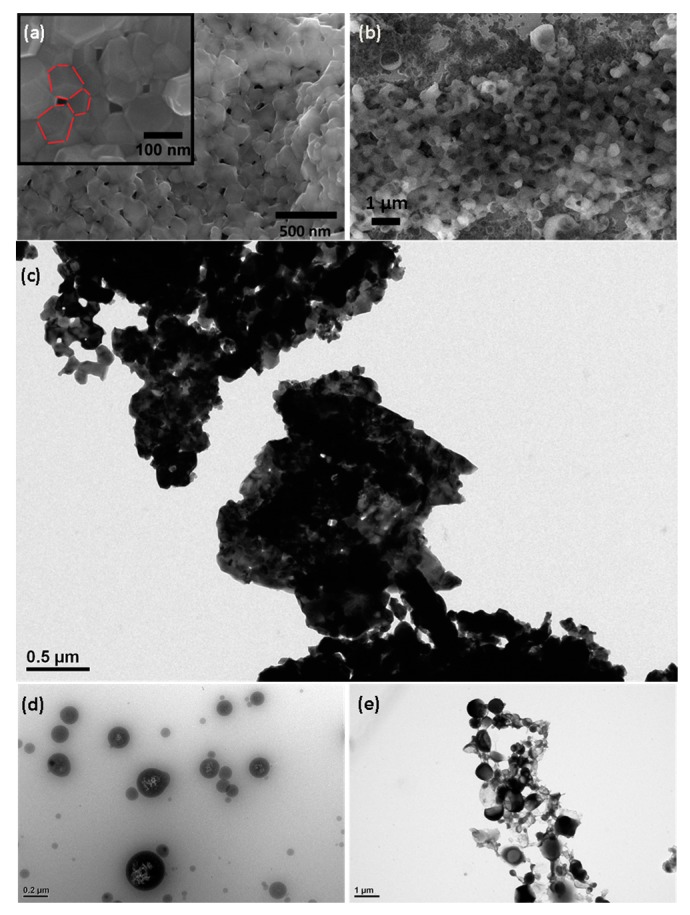
(**a**) SEM of Cu-ferrite NPs (in the inset, particles of *ca.* 70 nm are highlighted with red bars); (**b**) SEM of CA/5-LG/Cu-ferrite NCs; (**c**) TEM of Cu-ferrite NPs; (**d**) TEM of CA/5-LG NCs; and (**e**) TEM of CA/5-LG/Cu-ferrite NCs.

High resolution scanning electron microscopy revealed the presence of the Cu-ferrite nanoparticles and their distribution within the CA/5-LG NCs. The polygonal appearance of the Cu-ferrite nanoparticles is clearly shown in the inset in Figure 4a, which highlights also their tendency to aggregate in clusters, due to their spontaneous magnetic nature. Figure 4b shows the CA/5-LG/Cu-ferrite NCs, where the formation of the NCs is evident, which are much larger than the NPs of bare Cu-ferrite illustrated in Figure 4a. It seems from Figure 4b that the CA polymer with the lemongrass essential oil has well covered the Cu-ferrite NPs in accordance with the AFM images shown before (Figure 3). Similarly, the TEM image in Figure 4c shows the morphology of bare Cu-ferrite NPs that tend to aggregate due to their spontaneous magnetic nature. In Figure 4d, the TEM image of CA/5-LG NCs is shown, where it is clear that the NCs are spherical with a diameter up to 200 nm. In Figure 4e, the TEM image of CA/5-LF/Cu-ferrite NCs is illustrated, where a clear distinction between the NCs with and without Cu-ferrite NPs is shown. In Figure 4e, the morphology of the NCs is not as spherical as before: they tend to aggregate with each other; they have features that resemble tetragonal or cubic structures arising upon assembly with Cu-ferrites; and they have a larger diameter, more than 200 nm. Energy dispersive X-ray spectroscopy analysis (EDS) was used to confirm the Cu-ferrite nature on the CA/5-LG/Cu-ferrite NCs, and it is shown in Supplementary Materials Figure S1. The chemical composition of the Cu-ferrites on dried CA/5-LG/Cu-ferrite NCs was revealed with EDS as the Cu, Fe and O elements presented in the Cu-ferrite NPs (Figure S1). The % composition of the elements was O = 51.48%, Fe = 33.00%, Cu = 15.52%.

**Figure 5:**
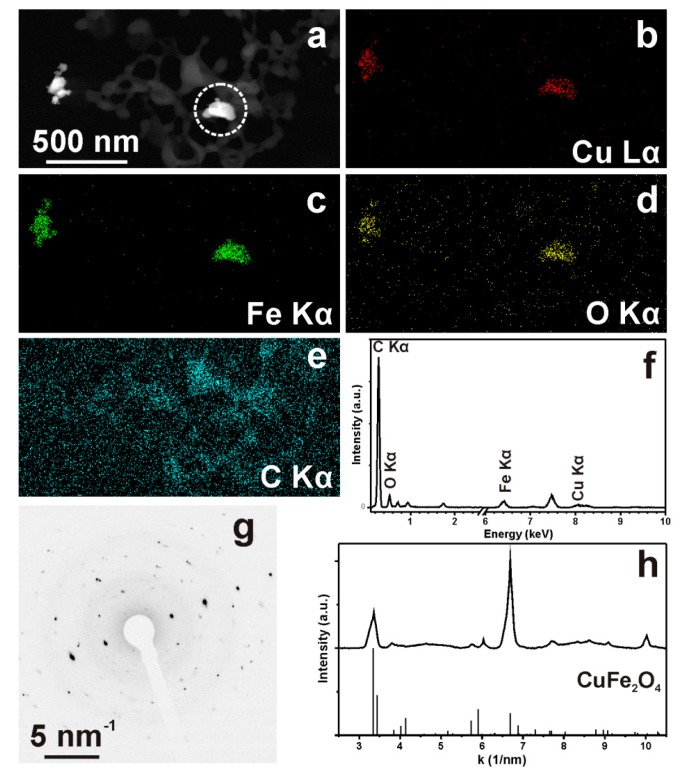
(**a**) HAADF-STEM and (**b**–**e**) corresponding STEM-EDS maps of Fe, Cu, O and C distribution over a region of the sample. The quantification of the (**f**) corresponding EDS spectrum gives atomic wt % Cu:Fe:O:C = 0.5:0.98:1.0:97.52; (**g**) SAED pattern from Cu-ferrite NPs and (**h**) corresponding azimuthal integration, compared to the database pattern for tetragonal CuFe2O4 (ICSD 188855).

In Figure 5, the HAADF-STEM and STEM-EDS data on a selected area of CA/5-LG/Cu-ferrite NCs dried on a grid are shown. We can see that the Cu-ferrites are localized in some areas around the polymeric capsules, and the SAED pattern proved their tetragonal structure. It was noticed that the combination of the vacuum inside the TEM together with the application of the electron beam (200-kV acceleration voltage) was creating instability of the capsules and was decomposing the structure of the cellulose acetate. In the solution, it is concluded from the DLS data shown before that the NCs preferentially remain as independent capsules without aggregating effects.

### 2.5. Magnetic Measurements

**Figure 6:**
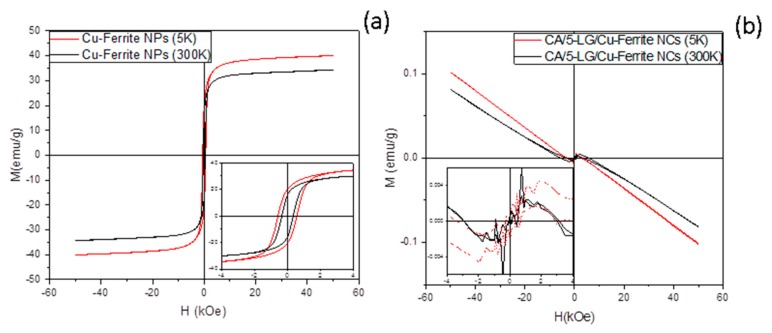
Magnetization curve of (**a**) Cu-ferrite nanopowder and (**b**) lyophilized CA/5-LG/Cu-ferrite NCs measured at 5 K and 300 K; the insets show a magnification of the corresponding hysteresis loop in the low field range.

Magnetization measurement was performed at 5 K and room temperature (300K) on bare and CA-encapsulated Cu-ferrite NPs (Figure 6). The magnetic behavior of the bare Cu-ferrite (Figure 6a) is typical of a system of ferro-/ferri-magnetic nanoparticles in the blocked state. The temperature dependence of the magnetization can be understood on the basis of the thermal fluctuation of the magnetic spins, while the decrease of the coercivity with temperature suggests the approaching of the nanoparticle systems to the superparamagnetic regime. The magnetization of saturation, reached for *ca.* 3 kOe, is consistent with literature data [21]. When Cu-ferrite nanoparticles are encapsulated with CA with and without LG (sample containing LG, CA/5-LG/Cu-ferrite NCs, shown in Figure 6b), the magnetic behavior of the composite is completely dominated by the diamagnetic contribution of the matrix, evidenced by the linear trend with the negative slope reported in Figure 6b. The very weak magnetic contribution from Cu-ferrite nanoparticles can be still observed below 2 kOe magnetic field intensities, as shown in the inset of the graph. The observed behavior is justified by the very low concentration of the Cu-ferrite NPs in the sample, which, from a very rough estimation obtained comparing the magnetization curves of Figure 5, is as low as 0.1–0.2 per thousand.

We assume that the low response of mixed CA/5-LG/Cu-ferrite NCs to an external magnetic field will reduce the possibility to use these NCs to direct them to a specific target-tissue for biomedical applications by means of an external magnetic gradient [12,24,25], but these NCs will be advantageous in other applications where antimicrobial drug delivery is needed without aggregating effects, such as skin and mucosal infections [8,26] or for other biomedical applications [27]. Furthermore, the low magnetic properties of CA/5-LG/Cu-ferrite NCs are advantageous, since Cu-ferrites have spontaneous magnetism and tend to cluster together; whereas such clustering can be avoided when polymeric NCs, such as the current CA, is involved, leaving the NCs apart and available for drug delivery applications.

### 2.6. Raman Spectroscopy

**Figure 7:**
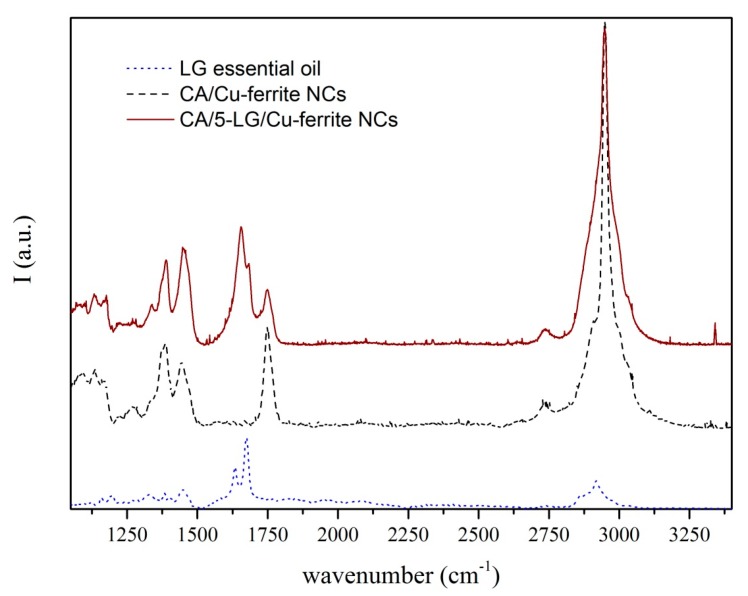
Raman spectra of LG essential oil (blue dots), CA/Cu-ferrite NCs (black dashed line) and CA/5-LG/Cu-ferrite NCs (brown line).

Raman spectra of CA/Cu-ferrite NCs, CA/5-LG/Cu-ferrite NCs and bare LG essential oil are presented in Figure 7. LG oil has two characteristic peaks at 1625 and 1670 cm^−1^, which are associated with C=C and C=O bonds arising from the two aldehydes of LG; neral and geranial [2,28]. These two peaks due to the C=C and C=O bonds appeared also in the CA/5-LG/Cu-ferrite NCs, but shifted at higher wavelengths, 1655 cm^−1^ and 1682 cm^−1^, respectively, due to hemi-acetal reactions [29,30,31] that take place between the aldehyde molecules in LG essential oil and the OH groups of the CA matrix. This hemi-acetal formation has been shown before for CA/5-LG NCs, but the C=C and C=O bonds appeared at slightly different wavelengths compared to the CA/5-LG/Cu-ferrite NCs; the reason for this shift should be the presence of Cu-ferrites that change the absorbance wavelength of the C=C and C=O bonds. 

### 2.7. UV-VIS Absorption Spectroscopy

**Figure 8:**
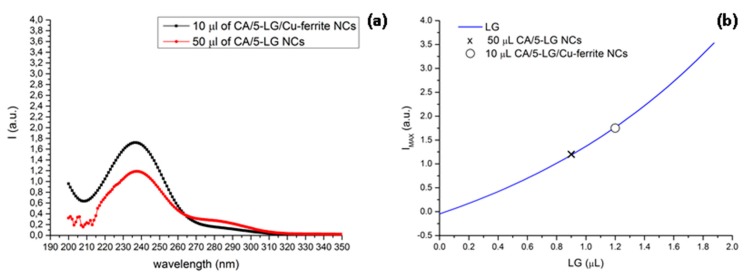
(**a**) UV-VIS absorption spectra of the 10-μL CA/5-LG/Cu-ferrite NC and 50-μL CA/5-LG NC solution and (**b**) the calibration curve and calculation of LG oil content in 10 μL CA/5-LG/Cu-ferrite and 50 μL CA/5-LG NCs.

Before proceeding with the antimicrobial analysis, it was necessary to evaluate the amount of LG present in 50 μL of CA/5-LG/Cu-ferrite NCs solution used for the antimicrobial analysis. UV spectra of 10 μL CA/5-LG/Cu-ferrite NCs and of 50 μL CA/5-LG NCs are shown in Figure 8a. The reason why we used 10 μL CA/5-LG/Cu-ferrite NCs was that the 50 μL CA/5-LG/Cu-ferrite NCs saturate the UV absorption signal. Moreover, UV spectra of bare LG essential oil were acquired with increasing amounts of LG to obtain a calibration curve of the intensity of the peaks at 238 nm *vs.* the LG volume, as shown in Figure 8b. To calculate the amount of LG in 10 μL CA/5-LG/Cu-ferrite NCs and of 50 μL of CA/5-LG NC solution, the intensity of the UV spectrum at the 240-nm peak was extrapolated on the calibration curve. UV analysis on 10 μL of CA/5-LG/Cu-ferrite NCs (Figure 7) showed that the content of LG inside the capsules was about 1.2 μL. Therefore, in 50 μL of the CA/5-LG/Cu-ferrite NCs that used for the antimicrobial experiment, as will be shown below, there were 6 μL or 5.322 μg of LG, which was higher than the amount of LG found inside the CA/5-LG NCs without Cu-ferrites published before (0.9 μL), even if the initial concentration of LG used to prepare the NCs was the same [8]. This showed that the presence of Cu-ferrite NPs enhanced the attachment and, hence, the concentration and stabilization of LG inside the CA/5-LG/Cu-ferrite NCs, a result that is in accordance with other work on different essential oils, vanilla, patchouli and ylang ylang [32], as well as Satureja hortensis [33] stabilized into iron oxide nanoparticles, with antimicrobial properties. To exclude the interference of Cu-ferrite and CA absorption in the wavelength of around 238–240 cm^−1^, UV spectra were acquired and are shown in Figure S2.

**Figure 9:**
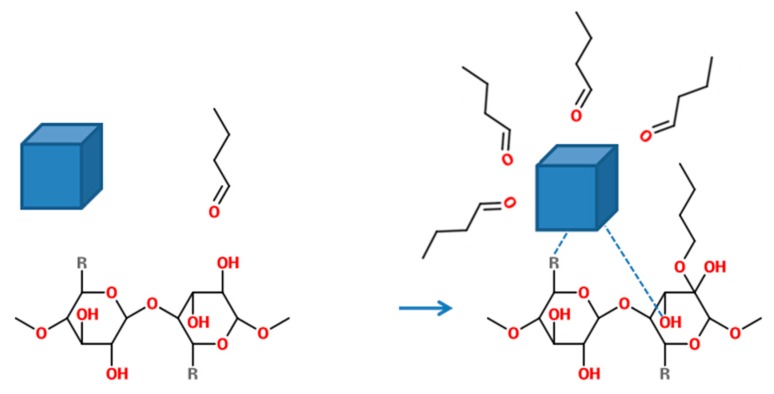
Molecular diagram of the reactions that can take place between aldehyde molecules, Cu-ferrite NPs and the hydroxyl group of the cellulose acetate ring to produce electrostatic bonds and hemiacetal bonds, respectively. The reactions between the Cu-ferrite NP with the acetyl group (R) and/or OH group of CA are also visible, where R is OC(=O)CH_3_.

A potential reaction that occurs between Cu-ferrite NPs and CA polymer is with the reaction of oxygen-rich groups, such as acetate groups or OH groups in the CA molecule, with Cu-ferrite. Similar reaction schemes between CA and iron [34] or copper [35] NPs have been shown before, where the O-rich groups of CA react with the metallic NPs. Taking into account also the mentioned UV studies, where a clear enhancement of the LG oil was present in the CA/5-LG/Cu-ferrite NCs, we also propose that some of the LG oil molecules, such as the prevailing aldehydes, react with the Cu-ferrite NPs, creating micelles with steric hindrance on them and not allowing the metallic NPs to aggregate. Such electro-steric stabilization has been also shown previously on Au NPs with carboxylic acids of Anacardium occidentale essential oils [36]. Therefore, a proposed mechanism of the NC’s formation is presented in Figure 9, where aldehyde molecules from essential oils can be bound to both OH groups of CA creating hemi-acetal bonds [8] and on metallic NPs electrostatically. It is also mentioned here that other polar molecules from LG essential oil, such as alcohols and acids, can also be bound to Cu-ferrite similarly. Furthermore, in Figure 9, the formation of metallic Cu-ferrite NPs with the acetyl or hydroxyl groups of CA is shown. To prove the existence of LG oil in both CA/5-LG NCs and CA/5-LG/Cu-ferrite NCs as bound to the CA polymer and/or Cu-ferrite NPs, thermogravimetric analysis (TGA) on lyophilized samples was performed as shown in Supplementary Materials Figures S3–S5, for samples with and without LG oil. As you can see, the weight loss of CA NPs without LG oil is very different from the NCs with LG oil, where the last ones started to loss their weight earlier due to the high volatility of LG oil at lower temperatures compared to the CA polymer.

### 2.8. Inductively Coupled Plasma Mass Spectrometry

ICP measurements of CA/5-LG/Cu-ferrite NC solution calculated the concentration of Cu and Fe, which was found to be 21.75 ppm and 28.3 ppm, respectively. The amounts of Cu and Fe found in 50 μL of CA/5-LG/Cu-ferrite NC solution used for the antimicrobial experiments were therefore 1.0875 and 1.415 μg, respectively; taking into account that ppm is expressed as mg/L.

### 2.9. Antimicrobial Tests

**Figure 10:**
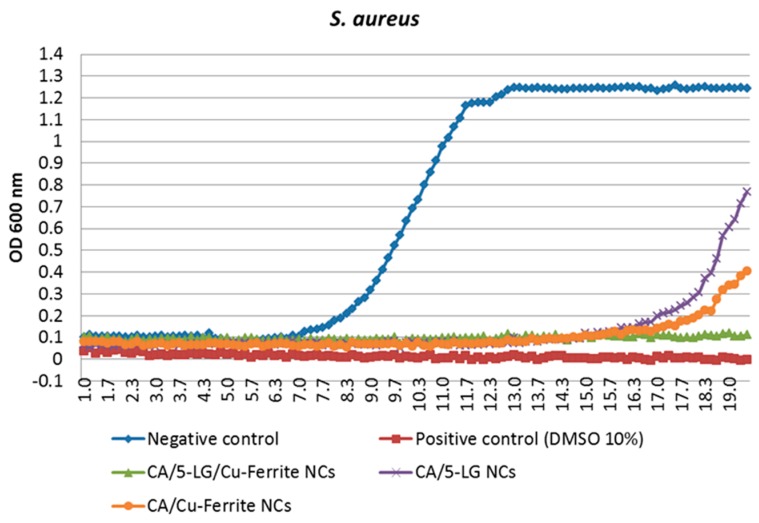
Antimicrobial activity of CA/5-LG, CA/Cu-ferrite and CA/5-LG/Cu-ferrite NCs.

The formed NCs were tested against *S. aureus* bacteria. We observe that bare CA NPs had no effect on bacteria, as expected from the inert nature of the CA polymer. Both CA/5-LG and CA/Cu-ferrite NCs were found to have very good antimicrobial activities when encapsulated into the CA polymer. In particular, the effect of Cu-ferrite was better than that of LG, as illustrated in Figure 10, with about 50% more efficacy. However, the combination of both LG and Cu-ferrite led to very efficient antimicrobial NCs, as the bacteria could not grow even after 20 hours of culture (Figure 10). This is a very important result, since it shows that a combination of organic and inorganic antimicrobial substances in a polymeric matrix can produce a nanomaterial with extremely good antimicrobial properties. Cu-ferrite is believed to have two positive effects on the antimicrobial activity; to provide an additional antimicrobial substance to the NCs and to stabilize and increase the LG content inside the NCs. Cu-ferrite NPs are supposed to adhere to bacterial cell wall and penetrate through the cell membrane; while copper ions cause destruction of the bacterial cell wall, degradation and lysis of the cytoplasm and, therefore, cell death [16,37,38]. Therefore, the excellent antimicrobial efficacy of the CA/5-LG/Cu-ferrite NCs was due to two reasons: first that the presence of Cu-ferrites led to an enhancement of LG essential oil concentration, as shown by UV studies, and second, the presence of Cu-ferrite inside the NCs change their morphology and chemistry, and thus, the presence of both antimicrobial agents LG essential oil and Cu-ferrites in the NCs increase the bacterial cell death.

## 3. Experimental Section

### 3.1. Materials

LG essential oil (100% pure) was purchased from Maitreya-Natura (Pesaro-Urbino, Italy). Cellulose acetate (CA) (acetyl content of 39.8%; MW 1/4 30 kDa) and acetone were purchased from Sigma Aldrich, Milano, Italy. Milli-Q water was used for the solvent/antisolvent method for the precipitation of the nanoparticles. *Staphylococcus aureus* (*S. aureus*) was purchased from ATCC (Washington, DC, USA). *S. aureus* was grown on Luria-Bertani (LB) medium, composed of: 1% tryptone (Sigma Aldrich), 0.5% yeast extract (Sigma Aldrich) and 1% NaCl (Sigma Aldrich).

### 3.2. Cellulose Acetate/Lemongrass Oil/Cu-Ferrite Nanocapsules Preparation

Cu-ferrite nanoparticles were synthesized by the citrate-gel auto-combustion technique using the source materials ferric nitrate (Fe(NO_3_)_3_·9H_2_O) and copper nitrate (Cu(NO_3_)_2_·3H_2_O). The metal nitrates were dissolved in 100 mL of distilled water and stirred for 15 min, and then, citric acid was added in the ratio of 1:1 to the metal nitrate solution. Ammonia droplets of a 33% concentration were added to adjust the pH of the mixture to 7. The solution was continuously stirred at 130 °C and then was transferred into xerogel. The dried gel burns in self-propagating combustion to form the ferrite nanostructure. The reaction causes a rapid release of gasses with a great mass loss leading to ferrite nano-powder formation. The Cu-ferrite nano-powder was then collected, and 0.1 g of it was inserted into 200 mL of Milli-Q water, which was placed in an ultrasonic bath (1 min at 40 KHz) prior to its use. Then, 100 mL of CA (10% *m*/*v*)/LG essential oil (5% *v*/*v*) was inserted drop-wise into the aqueous Cu-ferrite solution. This mechanism of the formation of NCs is described in a previous article [8], where CA and LG are chemically bonded, forming hemi-acetal bonds between the hydroxyl groups of the cellulose acetate and the aldehyde groups of LG essential oil. Cu-ferrite is supposed to be formed with the NCs by the aid of the hydrocarbon tails found in LG essential oil. Then, the precipitated NCs were filtered using Sartorius 389F filters and left for 4 h under nitrogen flow until the remaining acetone was evaporated. The number 5 before LG in CA/5-LG/Cu-ferrite NCs denotes the initial volume percentage of LG essential oil.

### 3.3. Dynamic Light Scattering

Dynamic light scattering (DLS) measurements were acquired using a Zetasizer Nanoseries from Malvern Instruments. The Z-average size was used in DLS, is a parameter also known as the cumulants mean, defined as the “harmonic intensity averaged particle diameter” and is hydrodynamic in nature, applicable to particles in dispersion. 

### 3.4. X-ray Diffraction

X-ray diffraction (XRD) patterns were recorded on a Rigaku SmartLab X-Ray diffractometer equipped with a 9-kW CuKα rotating anode (operating at 40 kV and 150 mA) and D\teX Ultra 1D detector set in X-ray reduction mode. The diffraction patterns were collected at room temperature in Bragg-Brentano geometry over an angular range 2θ from 15° to 85°, scan speed = 0.1°/min and step size = 0.02°. XRD data analysis was carried out using PDXL 2.1 software from Rigaku.

### 3.5. Atomic Force Microscopy

For atomic force microscopy (AFM) measurements, a Park Systems AFM instrument (XE-100, Suwon, Korea) was used in true non-contact mode. The images were acquired in air on an anti-vibration table (Table Stable TS-150) and within an acoustic enclosure. Single-beam silicon cantilever tips (PPP-NCHR-10) were used for the data acquisition with about less than a 10-nm nominal radius and a 42-N/m elastic force constant for high sensitivity. The resonance frequency was defined around 280 kHz. The scan rate was maintained at 0.1 Hz.

### 3.6. High Resolution Scanning Electron Microscopy, Energy Dispersive X-ray Spectroscopy and Transmission Electron Microscopy

High-resolution scanning electron microscopy (HRSEM) imaging was carried out using a JEOL JSM 7500FA (Jeol, Tokyo, Japan) equipped with a cold field emission gun (cold-FEG), operating at a 15-kV acceleration voltage. Compositional contrast was achieved using a retractable backscattered electron detector (RBEI), to better reveal the presence of Cu-ferrite within the CA-LG particle matrix. Energy dispersive X-ray spectroscopy (EDS) was performed using an Oxford X-Max 80 system with a silicon drift detector (SDD) having an 80-mm^2^ effective area of the detecting device. Samples for HRSEM were prepared by drop casting 5 µL of solution onto a silicon wafer. Transmission electron microscopy (TEM) images have been acquired with a JEOL JEM-1011 microscope, equipped with a tungsten thermionic electron source and operated at 100 kV of acceleration voltage. Samples were prepared by drop casting 20 μL of the solution onto carbon-coated copper grids. Selected-area electron diffraction (SAED) patterns and high-angle annular dark field-scanning TEM images (HAADF-STEM) images were acquired on a JEOL JEM-2200FS TEM (Schottky emitter, operated at 200 kV), equipped with a Bruker Quantax400 systems and a XFlash 5060 SDD detector for STEM-EDS analyses. For EDS analyses, the samples were deposited onto a C-coated Ni grid, and a holder without Cu parts was used, so as to allow for Cu quantification.

### 3.7. Magnetic Measurements

The magnetic properties of the bare and encapsulated Cu-ferrite nanoparticles, with and without LG, were investigated using a QuantumDesignMPMS SQUID magnetometer (QuantumDesign, San Diego, CA, USA) operating in the temperature range 2 K/300 K with an applied field up to 5 Tesla. Measurements were performed on the sample in solution and on lyophilized powder. The magnetic moment was measured as a function of the field cycling between +5 T and −5 T at 5 K and 300 K (room temperature). The recorded magnetic moments were normalized for the total mass of the sample.

### 3.8. Raman Spectroscopy

A Horiba Jobin-Yvon µRaman operating with a He-Ne laser source was used to study the molecular vibrations modes of bare LG oil, CA NPs and CA/5-LG/Cu-ferrite NCs. The wavelength of the laser radiation was 632.8 nm, and the objective used was a 100× with a slit aperture of about 200 µm.

### 3.9. UV-VIS Absorption Spectroscopy

A UV-VIS-NIR spectrophotometer by Varian (Cary 6000i) in double beam configuration was used to study the LG oil content in the CA/5-LG/Cu-ferrite NCs. First, a calibration curve was constructed by using bare LG essential oil in acetonitrile where the amount of the oil varied from 0.18 μL to 3.50 μL, as described analytically in a previous work [8]. To determine the amount of LG oil in CA/5-LG/Cu-ferrite NC solution, the measured intensity values at 240 nm were extrapolated to the calibration curve of bare LG. All of the UV measurements were done in acetonitrile solvent (3 mL).

### 3.10. Inductively Coupled Plasma Mass Spectrometry

To obtain the concentrations of Cu and Fe in the NCs, an ICP-OES spectrometer (iCAP 6300 DUO, ThermoFisher) was used. A volume (400 μL) of NCs was digested in 10% of aqua regia (HCl/HNO_3_ 3:1 (*v*/*v*)), incubated overnight at room temperature. Afterwards, Milli-Q water was added up to 10 mL and filtered first with an RC 0.45-μm filter to remove possible polymers in excess and lastly with a PTFE 0.45 filter. After calibration of the instrument, the most sensitive wavelength for Cu (224.7 nm) and Fe (238.2 nm) were considered.

### 3.11. Antimicrobial Tests

The biological properties of the produced NCs were tested against *S. aureus* (Gram-positive) bacteria. Sterile conditions were used, and the NCs were manipulated inside a Class II biohazard hood. For the antibacterial assays, cultures of *S. aureus* were grown overnight in LB medium in a 37 °C incubator. The cultures were then diluted to obtain 10^6^ CFUs/mL bacteria suspensions that were placed in sterile 96 well plates, 50 μL in each well. Eight replicates were produced for the tests. Immediately before starting the incubation, 50 μL of the NCs’ solution was added in each well. The bacterial growth was recorded measuring the absorbance at 600 nm every 10 min by using the microplate reader Fluo Star Optima (BMG LABTECH). The plates were maintained in culture conditions for the duration of the experiment (37 °C and double orbital shaking). Data were collected and elaborated with MARS DataAnalysis Sofware (BMG LABTECH). The negative control was then the LB medium, whereas for the positive control, we used 10% DMSO.

## 4. Conclusions

We showed that Cu-ferrite NPs were immobilized in CA/5-LG NCs, increasing the size of the final construct, CA/5-LG/Cu-ferrite NCs, to about 220–300 nm (from 150 nm of CA/5-LG NCs). AFM, SEM and TEM studies showed that Cu-ferrite presence in the CA/5-LG/Cu-ferrite NCs significantly changes the morphology of the NCs, making them larger, tetragonal and/or cubic as opposed to spherical and smaller in the absence of Cu-ferrite, *i.e.*, CA/5-LG NCs. The CA/5-LG/Cu-ferrite NCs did not aggregate into the solution due to their immobilization in the CA polymeric nanocapsules and their consequent magnetic hindrance, as found during the magnetic studies that revealed that CA/5-LG/Cu-ferrite NCs have very weak magnetic properties, which is advantageous for drug delivery applications when clustering effects due to the spontaneous magnetization of Cu-ferrites should be avoided. The antimicrobial tests showed that CA/5-LG and CA/Cu-ferrites have very good antimicrobial properties, but when both LG and Cu-ferrites were used simultaneously (CA/5-LG/Cu-ferrite NCs), the antimicrobial effect enhances significantly. The presence of LG oil is important for the stability of the NCs, since it also acts as a surfactant due to its polar groups and hydrophobic chains. Indeed, Cu-ferrite alone with CA produces NCs that tend to precipitate in a few hours, whereas the presence of LG produces stable NCs in water. The combination of active agents, LG essential oil and Cu-ferrites in the CA polymer produces stable NCs and a higher presence of LG essential oil in CA matrix compared to CA/5-LG NCs, as proven through the UV analysis. Cu-ferrite is believed to be attached to some of the oxygen-rich groups of CA, such as acetyl and hydroxyl groups, whereas LG essential oil was grafted at OH groups of CA with hemi-acetal formation and on the Cu-ferrite with electrostatic interactions. This work opens novel routes on the creation of NCs and NPs with the combination of organic and inorganic materials. The enhanced antimicrobial property obtained by the combination of the two active organic/inorganic components was tested on the *S. aureus* bacteria culture, the growth of which was reduced to zero, even after 20 h of incubation with CA/5-LG/Cu-ferrite NCs.

## Supplementary Materials

Supplementary materials can be accessed at: http://www.mdpi.com/1420-3049/21/4/520/s1.
