# Supplementary material for: Antimicrobial Lemongrass Essential Oil—Copper Ferrite Cellulose Acetate Nanocapsules
Source: Molecules. 2016 Apr 20;21(4):520. doi: 10.3390/molecules21040520 (PMC6273162; doi:10.3390/molecules21040520)
Supplement: Supplementary file 1 [file molecules-21-00520-s001.pdf]

Supplementary Materials: Antimicrobial Lemongrass Essential Oil–Copper Ferrite Cellulose Acetate Nanocapsules

Ioannis L. Liakos, Mohamed H. Abdelattif, Claudia Innocenti, Alice Scarpellini, Riccardo Carzino, Virgilio Brunetti, Sergio Marras, Rosaria Brescia, Filippo Drago and Pier Paolo Pompa

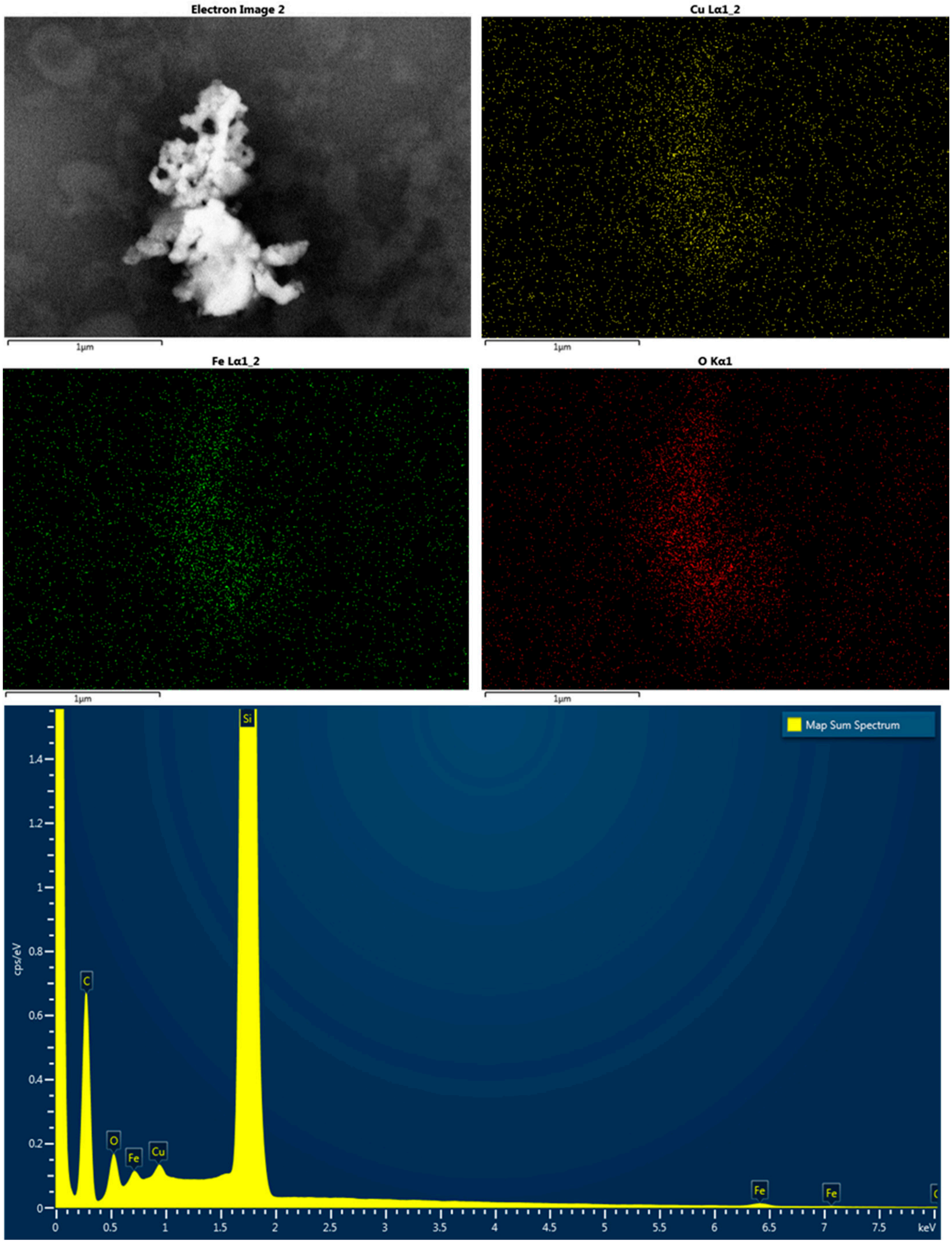

Figure S1. EDS mapping of Cu-ferrite NPs within the CA/5-LG/Cu-ferrite NC matrix; the first image on the top-left shows the analyzed region. Each map shows the distribution of Cu, Fe and O in correspondence with the cluster. The last image shows the corresponding EDS spectra. The scatter in the images is due to the localization of the Cu-ferrites, not only in the center of the image but also around, where the NCs are present.

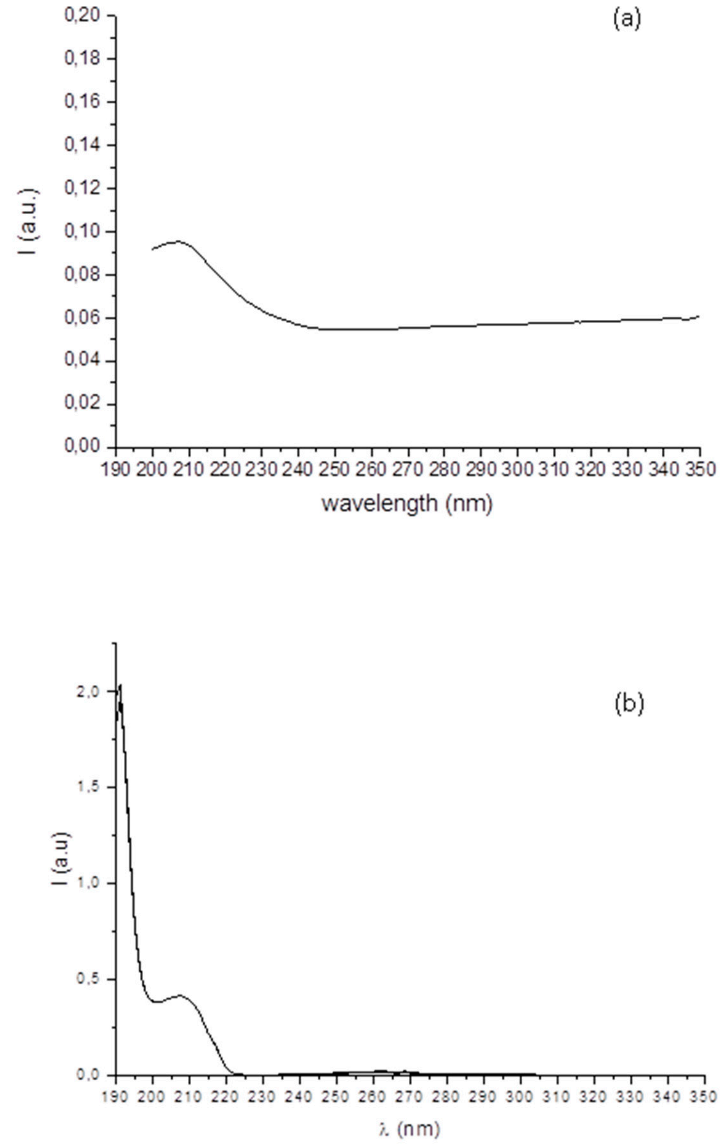

Figure S2. UV-VIS spectroscopy on (**a**) bare Cu-ferrite NPs and on (**b**) cellulose acetate NPs dissolved in acetonitrile.

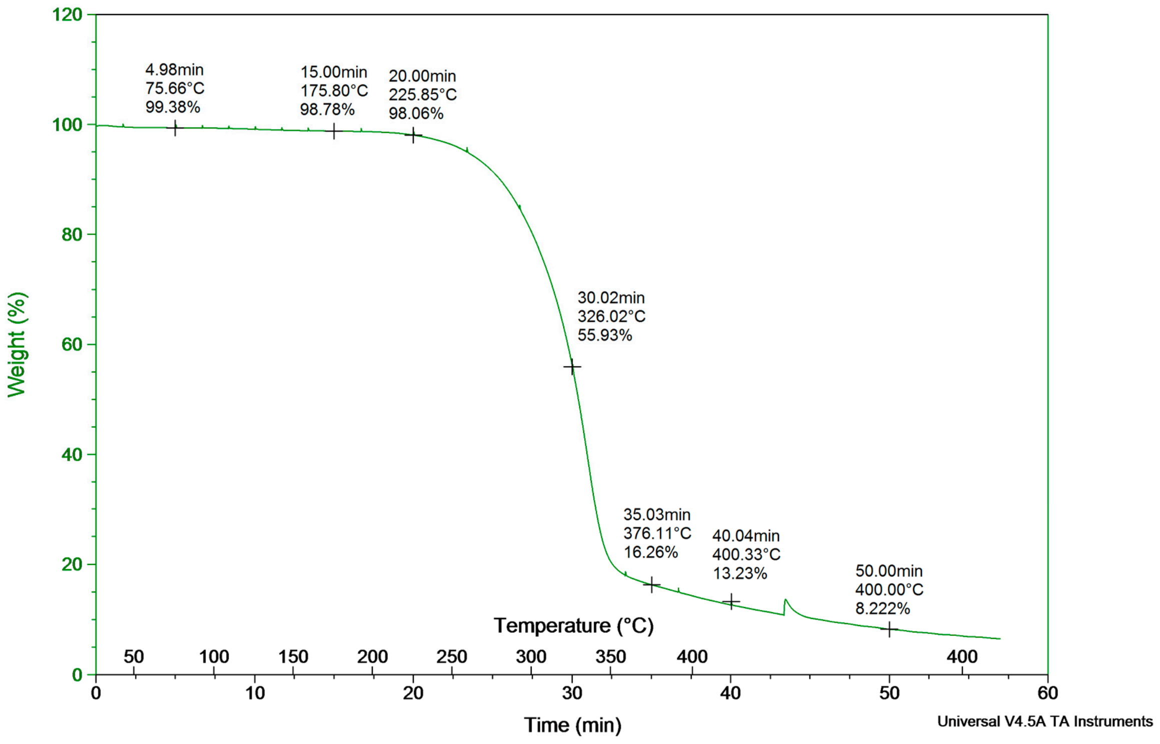

Figure S3. TGA CA NPs.

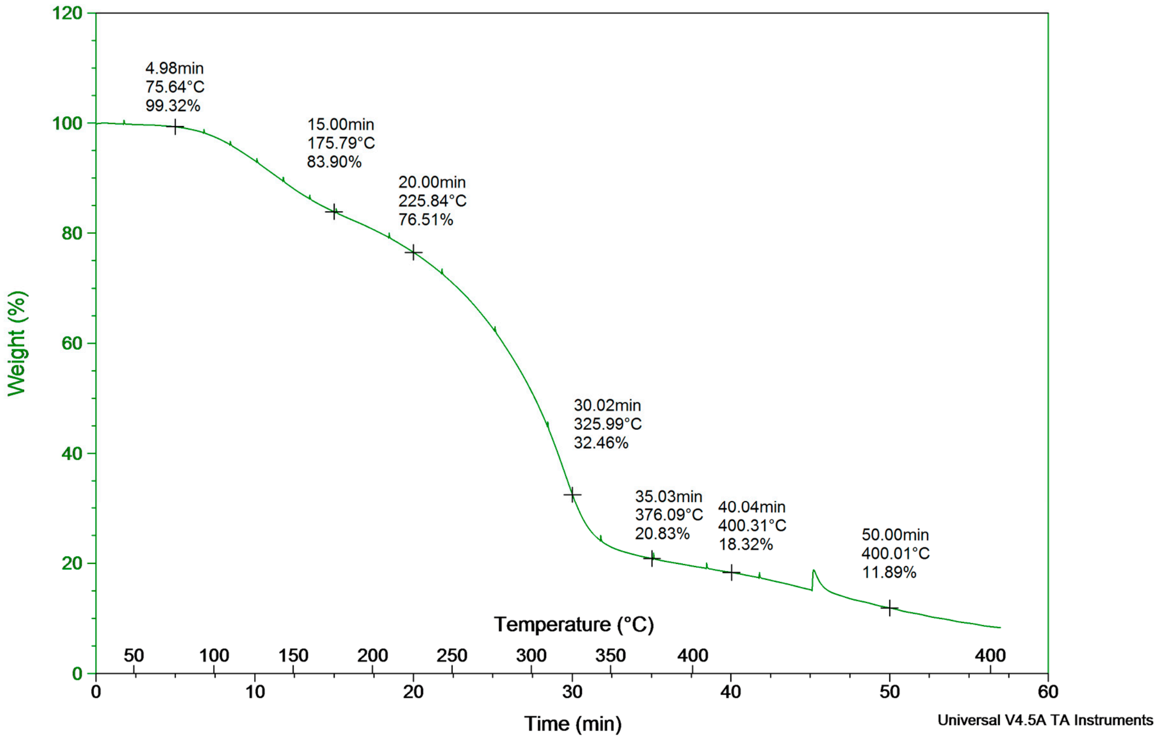

Figure S4. TGA CA/5-LG NCs.

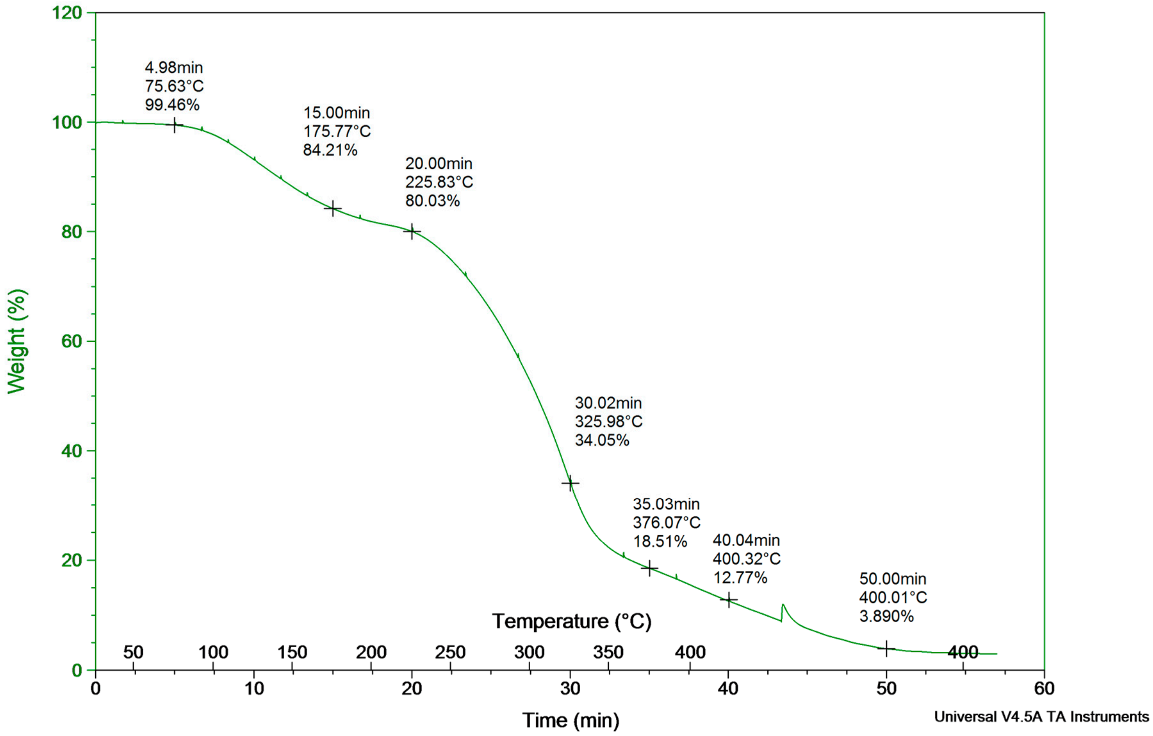

Figure S5. TGA CA/5-LG/Cu-ferrite NCs.
